# In-Depth Genomic and Phenotypic Characterization of the Antarctic Psychrotolerant Strain *Pseudomonas* sp. MPC6 Reveals Unique Metabolic Features, Plasticity, and Biotechnological Potential

**DOI:** 10.3389/fmicb.2019.01154

**Published:** 2019-05-24

**Authors:** Matias Orellana-Saez, Nicolas Pacheco, José I. Costa, Katterinne N. Mendez, Matthieu J. Miossec, Claudio Meneses, Eduardo Castro-Nallar, Andrés E. Marcoleta, Ignacio Poblete-Castro

**Affiliations:** ^1^Biosystems Engineering Laboratory, Center for Bioinformatics and Integrative Biology, Faculty of Life Sciences, Universidad Andres Bello, Santiago, Chile; ^2^Integrative Microbiology Group, Departamento de Biología, Facultad de Ciencias, Universidad de Chile, Santiago, Chile; ^3^Center for Bioinformatics and Integrative Biology, Faculty of Life Science, Universidad Andres Bello, Santiago, Chile; ^4^Computational Genomics Laboratory, Center for Bioinformatics and Integrative Biology, Faculty of Life Science, Universidad Andres Bello, Santiago, Chile; ^5^Centro de Biotecnología Vegetal, Facultad de Ciencias de la Vida, Universidad Andres Bello, Santiago, Chile; ^6^FONDAP Center for Genome Regulation, Santiago, Chile

**Keywords:** *Pseudomonas*, genome sequencing, aromatic compounds, poly(3-hydroxyalkanoates), low temperature, heavy metals, extremophile

## Abstract

We obtained the complete genome sequence of the psychrotolerant extremophile *Pseudomonas* sp. MPC6, a natural Polyhydroxyalkanoates (PHAs) producing bacterium able to rapidly grow at low temperatures. Genomic and phenotypic analyses allowed us to situate this isolate inside the *Pseudomonas fluorescens* phylogroup of pseudomonads as well as to reveal its metabolic versatility and plasticity. The isolate possesses the gene machinery for metabolizing a variety of toxic aromatic compounds such as toluene, phenol, chloroaromatics, and TNT. In addition, it can use both C6- and C5-carbon sugars like xylose and arabinose as carbon substrates, an uncommon feature for bacteria of this genus. Furthermore, *Pseudomonas* sp. MPC6 exhibits a high-copy number of genes encoding for enzymes involved in oxidative and cold-stress response that allows it to cope with high concentrations of heavy metals (As, Cd, Cu) and low temperatures, a finding that was further validated experimentally. We then assessed the growth performance of MPC6 on glycerol using a temperature range from 0 to 45°C, the latter temperature corresponding to the limit at which this Antarctic isolate was no longer able to propagate. On the other hand, the MPC6 genome comprised considerably less virulence and drug resistance factors as compared to pathogenic *Pseudomonas* strains, thus supporting its safety. Unexpectedly, we found five PHA synthases within the genome of MPC6, one of which clustered separately from the other four. This PHA synthase shared only 40% sequence identity at the amino acid level against the only PHA polymerase described for *Pseudomonas* (63-1 strain) able to produce copolymers of short- and medium-chain length PHAs. Batch cultures for PHA synthesis in *Pseudomonas* sp. MPC6 using sugars, decanoate, ethylene glycol, and organic acids as carbon substrates result in biopolymers with different monomer compositions. This indicates that the PHA synthases play a critical role in defining not only the final chemical structure of the biosynthesized PHA, but also the employed biosynthetic pathways. Based on the results obtained, we conclude that *Pseudomonas* sp. MPC6 can be exploited as a bioremediator and biopolymer factory, as well as a model strain to unveil molecular mechanisms behind adaptation to cold and extreme environments.

## Introduction

Microbial life thrives in extreme environments with very contrasting temperatures, ranging from 121°C in the thermal vents found deep in the ocean to -35°C in the permafrost of polar regions (Panikov and Sizova, 2007; Dick et al., 2013). Bacteria have been particularly successful in adapting to cold temperatures, since as much as 80% of Earth’s surface has a temperature below 5°C (Rodrigues and Tiedje, 2008). The Antarctic continent is one of the coldest, driest and most chemically extreme terrestrial environments to be inhabited by organisms. However, although hostile to most eukaryotes, it still houses a surprising diversity of microbes, indicating that such extreme conditions present no obstacle for microbial survival (Tindall, 2004). As one of the most poorly explored areas, this continent deserves further investigations at the microbial level and emerges as a substantially prospective region for the discovery of novel bacteria and bioactive metabolites (Bull and Stach, 2007). The upper layer tundra of the polar terrestrial environment, alternating between a frozen winter state and a thawed summer state, represents a very particular and challenging environment in which microorganisms must adapt their metabolism to work properly over a wide range of temperatures. Psychrophiles are microbes that can propagate below the freezing point of water (0°C) but that no longer replicate at temperatures above 20°C (Morita, 1975). A more versatile class of microorganisms are the so-called psychrotolerant or cold-adapted bacteria, as they display a wider growth temperature range (from -5°C to 40°C), with an optimal growth temperature that is usually above 25°C (Cavicchioli, 2016). In the last two decades, both classes of microorganisms have drawn great attention due to their unique molecular mechanisms to sustain life in harsh environmental conditions (D’Amico et al., 2006; De Maayer et al., 2014) as well as their potential for synthesizing valuable chemicals and proteins that have a broad array of industrial applications (Cavicchioli et al., 2011; Perfumo et al., 2018).

The advent of next generation sequencing (NGS) technologies has rapidly expanded our understanding of the genetic features of cold-adapted bacteria (Bowman, 2017). These features range from an elevated copy number of genes coding for cold shock proteins and factors involved in polysaccharide synthesis, to altered amino acid composition in enzymes toward an enhanced content of serine and polar residues for improving catalytic efficiency or enzyme turnover at low temperature (Georlette et al., 2004; Methé et al., 2005; de Bethencourt et al., 2015). Despite these advances, several unsolved questions persist, especially concerning to pathway functioning, energy-growth rate tradeoffs, cryoprotectants, and large protein stabilization components in heterotrophic bacteria. Recent studies also indicate that intracellular carbon storage compounds, e.g., Poly(3-hydroxyalkanoates) (PHAs), play a pivotal role in microbial resistance when exposed to temperature downshift (Ayub et al., 2009; Tribelli and López, 2011). Moreover, genetic elements involved in DNA repair (*recBCD* genes) have been shown to be essential during growth of *Pseudomonas syringae* at 4°C (Pavankumar et al., 2010). A growing body of evidence supports the notion that the successful lifestyle of psychrotolerant bacteria at low temperatures is orchestrated not only by one, but several molecular mechanisms and gene topologies that act synergistically to counteract the cellular insults provoked by ice formation, low nutrient availability, oxidative stress, and thaw-freezing cycles (Fondi et al., 2014; Koh et al., 2017; Mocali et al., 2017). Thus, it is imperative to pursue a system-level understanding of the adaptations allowing bacteria to thrive in cold environments.

Systems biology approaches to achieve a global understanding of microbial processes begin sequencing the genome of the organism under study, aiming to unveil its encoded functions and metabolic capabilities (networks). Reconstructing metabolic networks allows a better understanding of the relationship between the genotype and the molecular physiology of microorganisms (Francke et al., 2005). This is also a first step toward the generation of mathematical models at genome scale, which can be used to predict resulting phenotypes under various environmental conditions and genetic manipulations (Price et al., 2004). Today, integration of omics data, such as transcriptomics, proteomics, and metabolomics, into these metabolic models opens up the possibility of deciphering complex interactions among the various molecular levels which in turn could lead to the extraction of in-depth mechanistic knowledge of biological processes (Hyduke et al., 2013).

In this work, we focused our attention toward a versatile *Pseudomonas* sp. MPC6 strain that was recently isolated from Deception Island (Antarctica), which has the ability to grow at nearly the same rate and reach equal levels of biomass whether subjected to 4 or 30°C (Pacheco et al., 2019). In addition, it displays a high PHAs and exopolysaccharide substances (EPS) production capacity when grown on glycerol in batch cultures. Microbes of the genus *Pseudomonas* are well known for their high ability to colonize various ecological niches (Timmis, 2002) and resist adverse environmental conditions (Poblete-Castro et al., 2012). Moreover, they are currently being exploited as whole-cell biocatalysts for the production of a myriad of industrial (Poblete-Castro et al., 2017) and *trans* chemicals (Nikel and de Lorenzo, 2018) along with the bioconversion of man-made recalcitrant compounds to less toxic agents (Nikel et al., 2014), and the use of low cost substrates for the synthesis of biodegradable polymers such as polyhydroxyalkanoates (Borrero-de Acuña et al., 2019; Poblete-Castro et al., 2019).

In this respect, the unique phenotypes previously reported for the cold-adapted *Pseudomonas* sp. MPC6 strain (Pacheco et al., 2019) might be supported by distinctive groups of genetic elements that confer plasticity to the bacterium, allowing it to successfully thrive in such extreme environment. Using a combination of sequencing technologies (Illumina and PacBio), we sequenced and fully assembled the complete genome of *Pseudomonas* sp. MPC6 strain, performing a variety of genomic analyses that guided further experimental validations directed to ascertain the biotechnological potential and safety of this bacterium, as well as prompting new questions relating to molecular strategies and the lifestyle of psychrotolerant *Pseudomonas.*

## Materials and Methods

### Bacterial Strains and Growth Conditions

*Pseudomonas putida* KT2440 (DSM 6125), *Pseudomonas antarctica* (DSM 15318), *Pseudomonas aeruginosa* PAO1 (DSM 22644), *Pseudomonas syringae* pv. tomato DC3000 (ATCC BAA871), and the isolated Antarctic strains *Pseudomonas* sp. MPC6 were used throughout this study. The bacterial strains were stored in cryotubes at -80°C in 25% glycerol. To propagate the cells, each were plated onto Luria Bertani (LB) agar plates and further placed in an incubator for 24 h at 30°C, with the exception of *P. aeruginosa* which was cultured at 37°C. Liquid cultures were prepared by taking one colony from each plate and growing them in LB liquid media at 30°C, unless otherwise stated.

To assess the growth profile of *Pseudomonas* sp. MPC6 under different temperature conditions (ranging from 4 to 45°C), a colony from the LB plate was inoculated into a 50 mL flask with 10 mL of salt minimal medium (M9), consisting of the following chemicals (g/L): Na_2_HPO_4_ ⋅ 7H_2_O, 12.8; KH_2_PO_4_, 3; NaCl, 0.5; NH_4_Cl, 1. This medium was sterilized (autoclaved) and supplemented with filter-sterilized MgSO_4_ ⋅ 7H_2_O (0.12 g/L) and 1 mL/L of a trace element solution consisting of (g/L): CaCO_3,_ 2.7; FeSO_4_ ⋅ 7H_2_O, 6; MnSO_4_ ⋅ H_2_O, 1.16; ZnSO_4_ ⋅ H_2_O, 2.0; CuSO_4_ ⋅ 5H_2_O, 0.33; H_3_BO_3_, 0.08_;_ CoSO_4_ ⋅ 7H_2_O, 0.37). Glycerol (4 g/L) was used as the sole carbon and energy source. The MPC6 strain was then grown at various temperatures in a rotary shaker spinning at 160 rpm (Ecotron, INFORS HT, Switzerland).

All cultures started with an initial optical density OD_600_ of 0.05, by calculating the specific volume of preinoculum required, where the cells were grown in 500 mL baffled shaking flasks containing 100 mL of M9 medium and (4 g/L) glycerol at 30°C and in constant agitation at 180 rpm.

The MPC6 cells were cultured at a temperature of 0°C using a 1 L vessel (0.8 L working volume) jacketed bioreactor (Labfor5, INFORS HT, Switzerland), where a coolant fluid was passed through the jacket of the bioreactor employing a chiller (Julabo F500, Germany). Using a mass flow controller, the aeration rate was set at 0.4 L/min. By controlling the agitation speed, the oxygen tension was perpetually kept above 20% (800 rpm). The pH value was not controlled in order to make it comparable with flask experiments.

For PHA synthesis, *Pseudomonas* sp. MPC6 cells were inoculated in M9 medium supplemented with the desired carbon source and incubated overnight in an incubator shaker (Ecotron, INFORS HT, Switzerland) set at 160 rpm and 30°C. The final culture was carried out in a 500 mL shaking flask with 100 mL of M9 salt medium and different C substrates in a shaker set at 30°C and 160 rpm. The starting OD_600_ of the culture was always 0.05 and the length of the cultivation was 96 h for all tested conditions, where cells were harvested for PHA quantification.

### Genome Sequencing, Assembly and Annotation

Genomic DNA was extracted from an overnight culture of *Pseudomonas* sp. MPC6 grown at 30°C in LB medium, using the GeneJET Genomic DNA Purification Kit (Thermo Fisher Scientific), following the manufacturer’s guidelines. Genome sequencing was performed using both illumina MiSeq (Illumina Inc., San Diego CA, United States) and PacBio RS technologies. For illumina sequencing, the library was prepared using TruSeq DNA (Illumina Inc.) following the manufacturer’s instructions. We constructed a library of 600bp (index A007; CAGATC) and it was sequenced on a MiSeq Platform using MiSeq v3 Reagent Kit 600 cycles (2 × 300 bp paired-end), obtaining 5,215,528 single reads (Q30 > 83.16%). For PacBio sequencing, the library was prepared using a SMRTbell template preparation system, and then was run in a SMRT Cell V3, using the DNA Polymerase Binding Kit P6 and the DNA Sequencing Reagent 4.0 v2, obtaining 45,225 long reads comprising a total of 500,918,948 bp (N50 = 20,242 bp). Before assembly, the set of short reads was trimmed by quality using the tool Trimmomatic (Bolger et al., 2014). Genome assembly was performed by combining both the illumina and PacBio reads using Unicycler v0.4.8-beta in the hybrid assembly mode (Wick et al., 2017). Assembly progression and resulting genome were evaluated through visualization of assembly graphs generated at each stage of the Unicycler pipeline, using Bandage (Wick et al., 2015). The assembled replicons were annotated using the Prokaryotic Genome Annotation Pipeline available from NCBI as part of the GenBank submission process. For genomic analyses, additional strategies to perform further gene annotation and classification were conducted using the tools BLAST (Altschul et al., 1997), Prokka (Seemann, 2014), PHAST (Zhou et al., 2011), oriTfinder (Li et al., 2018), and Roary (Page et al., 2015).

### Phylogenetic Analysis of MPC6 and Other *Pseudomonas* Strains

The distance tree showing the phylogenetic relationships between MPC6 and other *Pseudomonas* strains at a sub-species resolution was calculated from a multiple sequence alignment generated using a core genome MLST approach, as described below. First, we used the tool Roary to compare the sequence of 39 *Pseudomona*s chromosomes (including MPC6) in order to identify a set of genes encoding proteins sharing 90% or more sequence identity among all the strains. A total of 45 protein coding genes meeting this criterion were selected for this analysis. The sequence of the encoded proteins was extracted from each strain and aligned using MUSCLE (Edgar, 2004). Then, the 45 alignments obtained were trimmed to remove the non-informative stretches using gblocks (Castresana, 2000), and concatenated using seqotron (Fourment and Holmes, 2016). The resulting concatenated alignment was used to infer a maximum likelihood distance tree by means of RAxML v8.2.X (Stamatakis, 2014), set to automatically determine the best-scoring protein substitution model, and to perform a bootstrap analysis involving 100 replicates. This procedure was repeated three times varying the seed parameters. The tree showing the best likelihood score was plotted and mid-point rooted using FigTree v1.4.3 (Rambaut, 2009).

### Metabolic Reconstruction

The complete genome sequence of strain MPC6 (Fasta format) was loaded onto ModelSEED^1^ where a model in SBML format (Systems Biology Marked Lenguaje) was created. This model was imported using COBRA Toolbox (Schellenberger et al., 2011) under Matlab^®^ (R2012a, Mathworks, United States). Using Paint4net for COBRA, the metabolic pathways were visualized and confirmed. Finally, the metabolic pathways of the central carbon metabolism, aromatics, fatty acid synthesis and degradation were drawn using Microsoft Visio Software.

### Virulence and Antimicrobial Resistance Profile Determination From Genomic Data

The identification of virulence-related genes among different *Pseudomonas* genomes was performed using the VFanalyzer pipeline available in the Virulence Factors of Pathogenic Bacteria (VFDB) database (Chen et al., 2015), as well as the gene classification tools available from the PATRIC database and analysis resource center (Wattam et al., 2016). For this, all the genomes included in the comparison were re-annotated using the annotation tool included in each platform. The lists of categorized genes were used to construct heatmaps reflecting the number of genes of each category found in each strain, compared to the total number of known genes for that category. Besides, the identification of antibiotic resistance genes in MPC6 and other *Pseudomonas* strains was performed using PATRIC and the Comprehensive Antibiotic Resistance Database (CARD) (Jia et al., 2016) tools. For heatmap construction, the predicted genes were grouped into the different categories and counted. The 0% was defined as complete absence (count = 0, color = white) while 100% (color = black) was the highest count for each category among the strains being compared.

### Antimicrobial Susceptibility Testing

Antimicrobial susceptibility determination was performed using a modified version of the EUCAST disk diffusion assay. Briefly, Mueller-Hinton agar plates were inoculated with a cotton swab previously immersed in a suspension of approximately 1 × 10^8^ bacterial cells/mL of the strain to be tested in sterile PBS. After 15 min, disks comprising different antibiotics (Oxoid) were evenly applied over the inoculated plates using an Oxoid disk dispenser, incubating them at 30°C during 16–20 h. The size of the growth inhibition halo was recorded for the different antibiotics and strains tested. The following disks were used: amikacin 30 μg/mL, ciprofloxacyn 5 μg/mL, colistin 10 μg/mL, clindamycin 2 μg/mL, erythromycin 15 μg/mL, cefepime 30 μg/ mL, Fosfomycin/trometamol 200 μg/mL, linezolid 30 μg/mL, meropenem 10 μg/mL, rifampicin 5 μg/mL, sulphamethoxazole/ trimethoprim 25 μg/mL, tigecycline 15 μg/mL, piperacillin/ tazobactam 110 μg/mL, and vancomycin 30 μg/mL.

### Minimal Inhibitory Concentration Assay

Minimal inhibitory concentration (MIC) of *Pseudomonas* sp. MPC6 was tested on various metal concentrations, ranging from 1 to 500 mM in LB liquid medium. The compounds used for the assay were CdCl_2_, CuSO_4_, HgCl_2_, NaAsO_2_, Na_2_HAsO_4_. A specific volume of grown cells was inoculated in 200 mL Erlenmeyer shaking flasks with 50 mL LB medium and propagated for 24 h at 30°C and 160 rpm in a rotary shaker. The MIC values for cadmium, mercury, copper, arsenate, and arsenite, the lowest metal concentration arresting visible cell growth, were recorded. Colony forming units displaying a reduction of more than 99.9% in bacterial growth when compared with controls were also considered MIC. These were measured by streaking a series of dilutions of *P. putida* cells onto LB agar plates and incubated in an oven at 30°C for 24 h.

### Biomass Quantification

Cell growth was evaluated by measuring OD_600_ using a spectrophotometer (UV-Vis Optizen 3220UV, Korea). The cell dry weight was determined gravimetrically after harvesting the cells from 10 mL of culture broth through centrifuging for 10 min at 4°C and 9,000 × *g* (Eppendorf 5810 R, Hamburg, Germany) in pre-weighed tubes. The cell pellet was washed once with distilled water, and then dried at 100°C until obtaining a constant weight.

### PHA Characterization and Quantification

Methanolysis of lyophilized cell dry mass (5–10 mg) was introduced in sealed tubes containing 2 mL chloroform, 2 mL methanol, 15% (v/v) H_2_SO_4_ and 0.5 (mg/mL) 3-methylbenzoic acid. The tubes were incubated for 4 h at 100°C in a thermoblock. After the tubes reached room temperature, 1 mL of miliQ water was mixed with the reaction solution, and vigorously agitated for 1 min. The mixture was then transferred to a Falcon tube (15 mL) and centrifuged for 10 min at 6,000 *g*. The lower part of the biphasic solution, containing the methyl esters of the biopolymer, was separated and analyzed via gas chromatography coupled to mass spectrometry (YL6900, Young Instruments, Korea) using the methodology previously described by Borrero-de Acuña et al. (2019). Briefly, a calibration curve was created using Polyhydroxybutyrate P(3HB) from Sigma-Aldrich and a purified medium-chain-length-PHA synthesized in a previous work (Oliva-Arancibia et al., 2017) to interpolate data samples. Once the retention time of the peaks were contrasted with the standards, their chemical structures were characterized based on the resulting mass compatibility (NIST 17 Mass Spectral library). The percentage of PHA in the cell was defined as the amount of the biopolymer divided by the total cell dry mass and multiplied by 100.

### Amino Acid Sequences Alignment and Phylogenetic Analysis of the PHA Synthases Encoded in the Genome of Strain MPC6

We found five PhaC-like polyhydroxybutyrate (PHA) synthase-coding genes in the *Pseudomonas* sp. MPC6 genome (based on Prokka annotation). To characterize them, its sequence was compared with PhaC sequences from different bacterial species retrieved from NCBI database. The following protein sequences (and its respective coding nucleotide sequence) were used: AAM63409.1 and AAM63409.1 (*Pseudomonas putida* KT2440); ACK57560.1 and ACK57562.1 (*Pseudomonas fluorescens*); BAA36200.1 and BAA36202.1 (*Pseudomonas* sp. 61-3); WP_003115652.1 (*Pseudomonas aeruginosa* PAO1); WP_004060157.1, WP_004060696.1, and WP_004056138.1 (*Haloferax mediterranei*); WP_013055939.1 (*Bacillus megaterium*); and WP_011615085.1 (*Cupriavidus necator*). These sequences, along with the five *Pseudomonas* sp. MPC6 PhaC-like sequences, were compared through multiple protein sequence alignments using MUSCLE v3.8.31 (Edgar, 2004), which were visualized using MView v1.63. In order to infer the phylogenetic relationship among all the PhaC sequences, we performed a multiple alignment of the coding nucleotide sequences guided by the protein sequence alignment of all PhaC sequences using TranslatorX v1.1 (Abascal et al., 2010). Next, we evaluated the best substitution model for the PhaC sequences using jModelTest2 v2.1.10 (Darriba et al., 2012), and used MrBayes v3.2.6 (Ronquist et al., 2012) to infer the phylogenetic tree, which we visualized using FigTree v1.4.4 (Rambaut, 2009).

## Results

### Genome Sequence of the Antarctic *Pseudomonas* sp. MPC6 Strain

*Pseudomonas* sp. MPC6 is a bacterial strain isolated from a soil sample collected at Deception Island (South Shetland archipelago, Antarctica), during the 53rd Chilean Scientific Antarctic Expedition (ECA53) in January–February 2016. It has the unique ability to produce high amounts of biomass, extracellular polysaccharides, and novel types of polyhydroxyalkanoates (PHAs) over a wide range of temperature, from 4 to 30°C (Pacheco et al., 2019). Given its outstanding features, we decided to perform a deep genomic and phenotypic characterization of this extremophile to get a better understanding of its metabolic properties and biotechnological potential.

**FIGURE 1:**
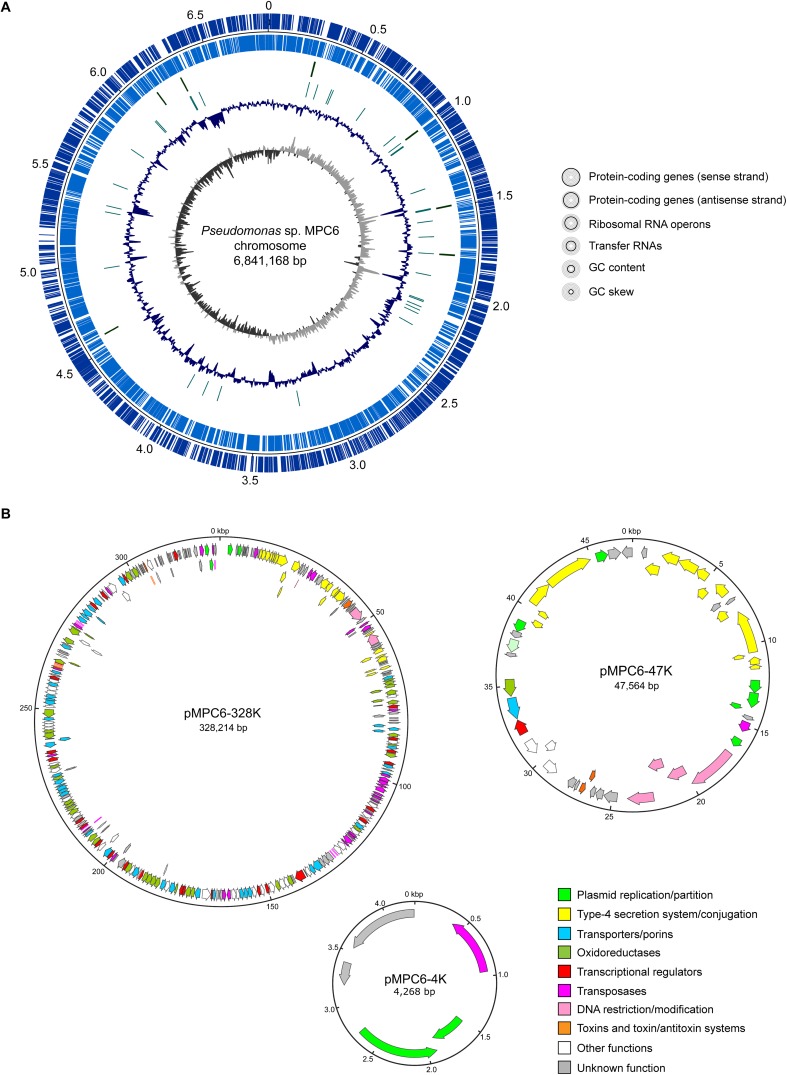
Circular maps showing the main features of the *Pseudomonas* sp. MPC6 chromosome and the three plasmids that compose its genome. **(A)** Chromosome map showing the protein coding genes in both strands, the genes coding for ribosomal and transfer RNAs, the GC content and the GC skew. **(B)** Map of the plasmids pMPC6-328K, pMPC6-47K, and pMPC6-4K. The genes identified inside each plasmid were colored according to its predicted function.

In order to obtain the complete genome sequence of this isolate, its genomic DNA was extracted and sequenced using both Illumina and PacBio technologies. After quality trimming, the short and long reads were combined to perform a hybrid assembly, using the tool Unicycler. A total of 4 closed circular contigs were assembled, giving a total genome size of 7,221,214 bp (Supplementary Table S1). The mean coverage was 114X, calculated as the sum of the sequenced bases that were incorporated into the hybrid assembly divided by the total genome size. The largest contig matched to the chromosome (6,841,168 bp; accession CP034783), showing a GC content of 59.96% (Figure 1A). After annotation, a total of 6,330 protein-coding genes were identified, as well as 69 tRNA-coding genes, 1 transfer messenger RNA-coding gene, and 22 rRNA-coding genes (corresponding to 7 full copies of the rRNA operon). No CRISPR loci nor integrated prophages were found across the chromosome.

Among the other circular contigs, three plasmids lacking full-length homologs in the NCBI database were identified (Figure 1B). The biggest plasmid (328,214 bp) named pMPC6-328K (accession CP034782) comprises 326 CDS and seems to be conjugative, since it harbors a set of genes encoding a putative type IV secretion system (T4SS), including a relaxase protein (ELQ88_00595; MobA homolog) and a T4SS coupling protein (ELQ88_00455; TrbC homolog), which correspond to key factors involved in conjugative transfer of plasmid DNA through this kind of systems in Gram-negative bacteria. However, no transfer origin (oriT) could be detected and some genes from this T4SS seem to be interrupted by insertion sequences, raising the question of whether this plasmid is actually mobilizable through conjugation. In addition, several other putative insertion sequences and transposase-coding genes were found in different regions of this plasmid, suggesting that these elements have a high impact on the evolution of this isolate. Additionally, pMPC6-328K encodes more than 40 putative transporter proteins, and a similar number of putative oxidoreductases, likely involved in the metabolism and uptake of different compounds and divalent metals, including L-carnitine, phenoxybenzoate, succinilbenzoate, gamma-aminobutyraldehyde, putrescine, betaine, glycine, manganese, and zinc. In addition, this plasmid encodes nearly 25 transcriptional regulators, as well as two toxin/antitoxin systems likely selecting against the loss of the plasmid. The second largest plasmid (47,564 bp) named pMPC6-47K (accession CP034781) harbored 51 CDS and also seems to be conjugative, comprising genes coding for a T4SS (different than that from pMPC6-328K) including a relaxase and a coupling protein (ELQ88_00260 and ELQ88_00255, respectively), although no oriT could be identified. Furthermore, pMPC6-47K encodes proteins potentially involved in L-tartrate and oxalacetate uptake and metabolism, in a type-1 restriction modification system, and in a RelE/ParE-like type II toxin-antitoxin pair (also different than that carried by pMPC6-328K). The third plasmid identified (4,268 bp) was named pMPC6-4K (accession CP034780), and carries 4 CDS, including a *repB* homolog likely involved in plasmid replication (ELQ88_00015), a gene encoding a putative serine recombinase (ELQ88_00005), and two hypothetical proteins with no predicted function.

### Phylogenetic Relationships Among *Pseudomonas* sp. MPC6 and Other Members of This Genus

We exploited the genomic information to phylogenetically classify *Pseudomonas* sp. MPC6. First, the assembled chromosome was analyzed with the “Identify species” tool based in ribosomal multilocus sequence typing (rMLST), available as part of the of the Bacterial Isolate Genome Sequence Database (BIGSdb) (Jolley and Maiden, 2010). Only low confidence assignments were obtained, where MPC6 was identified as *Pseudomonas mandelii* (42% support), as *Pseudomonas silesiensis* (28% support), and as *Pseudomonas lini* and *Pseudomonas prosekii* with a 14% support. This strongly suggests that MPC6 strain is part of a new species from the genus *Pseudomonas*.

**FIGURE 2:**
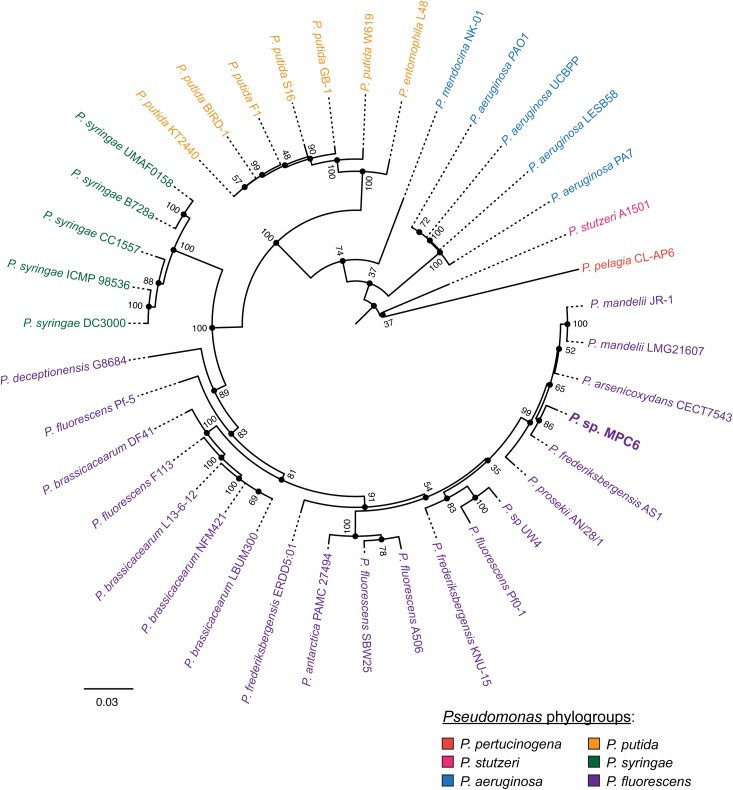
Phylogenetic relationships between *Pseudomonas* sp. MPC6 and other strains from this genus. The distance tree was calculated in base to a core genome multilocus sequence analysis as described in the “Materials and Methods” section. The different colors indicate the main phylogroups proposed to compose the genus *Pseudomonas* (Peix et al., 2018). The numbers aside each node indicate the bootstrap support calculated from 100 replicates. A complete description of the strains used to construct this tree is provided in the Supplementary Table S2.

Next, we performed a core genome multilocus sequence-based phylogenetic analysis to explore with higher resolution the relationships between MPC6 and 38 completely sequenced strains representative of a variety of species belonging to this genus, which are from diverse geographical origins and environmental conditions, virulence, phenotypic traits and time of isolation (see the Supplementary Table S2 for the full list of strains). To this end, we first used the tool Roary to determine the set of protein coding genes common to all the strains (90% amino acid sequence identity cutoff). Surprisingly, only 45 genes met the mentioned criteria, indicating that the strains included in the comparison, although part of the same genus, have highly diverse genomes. Next, we generated individual alignments of the selected 45 gene products from the 39 strains, filtered them to remove gaps and non-informative regions, and then concatenated them generating a final alignment encompassing a total of 10,965 amino acid residues. The concatenated alignment was used to infer a maximum-likelihood distance tree using the RAxML tool, set up to perform a bootstrap support analysis comprising 100 replicates (Figure 2). The analyzed strains clustered in agreement with classifications performed in previous works, covering several of the main species phylogroups proposed to compose the genus *Pseudomonas* (Peix et al., 2018), including *P. pertucinogena*, *P. stutzeri*, *P. aeruginosa*, *P. putida*, *P. syringae*, and *P. fluorescens*. *Pseudomonas* sp. MPC6 belonged to the *P. fluorescens* phylogroup, which was the most represented and diverse inside the sample, comprising the higher number of species. Inside this phylogroup, the MPC6 closest relative was *P. frederiksbergensis* AS1, although both strains formed separate branches in the tree (86% bootstrap support). Notably, AS1 strain was isolated from an arsenic contaminated site in Gwangju, South Korea, a generally rainy and warm region with temperatures over 30°C during summer, a very contrasting situation compared to Deception Island in Antarctica, from where MPC6 was isolated. Other close neighbors forming a wider supported clade along with MPC6 include *P. mandelii* LMG21607, *P. mandelii* JR-1, *P. arsenicoxydans* CECT7543, previously grouped inside the *P. mandelii* subgroup (Gomila et al., 2015), and more distantly related *P. prosekii* AN/28/1. These strains were also isolated from very diverse geographical locations and environments including natural mineral waters in Gyeongsan (South Korea), the Atacama Desert (Chile), and the James Ross Island (Antarctica) (Supplementary Table S2).

### Metabolic Reconstruction of *Pseudomonas* sp. MPC6

**FIGURE 3:**
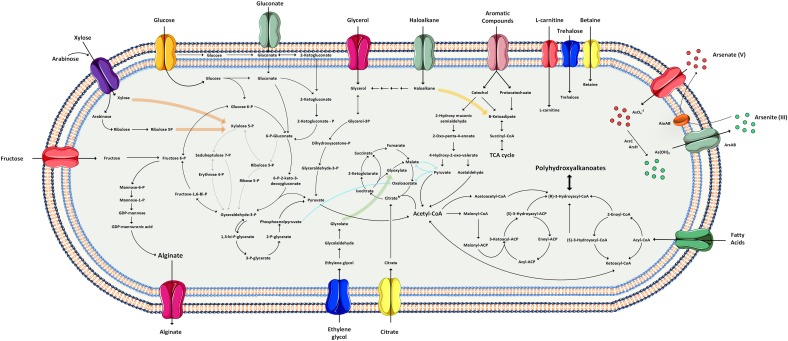
Reconstruction of metabolic pathways of the Antarctic *Pseudomonas* sp. MPC6 based on genomic information.

To get insight into the metabolic capabilities of *Pseudomonas* sp. MPC6, we reconstructed the central carbon metabolism, aromatics degradation routes and their connections with the synthesis of exopolysaccharides, unsaturated fatty acids, and Poly(3-hydroxyalkanoates) (PHAs) (Figure 3). As most bacteria of the genus *Pseudomonas*, the Antarctic microbe MPC6 does not possess the 6-phosphofructokinase enzyme (PFK) that plays a pivotal role for the conversion of sugars via the Embden–Meyerhof–Parnas (EMP pathway), a process also known as glycolysis. This suggests that most of the carbon conversion of glucose, fructose, gluconate, and polyols goes through the Entner–Doudoroff pathway (ED), a significant route that empowers environmental bacteria with high tolerance to oxidative stress and has a low protein cost (Chavarría et al., 2013; Chen et al., 2016). One remarkable feature of the MPC6 strain is its capacity to metabolize C5 carbon sugars such as xylose and arabinose. Examination of the MPC6 genome revealed that xylose can be converted to xylulose via isomerization (XylA enzyme), which is subsequently transformed to xylulose 5-phosphate (xylulokinase encoded by *xylB*), a compound that directly funnels the pentose phosphate pathway (PPP) (Köhler et al., 2015). We did not find any protein-coding genes of the Weimberg and Dahms pathway, which has been previously related to xylose metabolism. Moreover, arabinose can also be catabolized via the isomerase and oxo-reductive pathway. The genome of *Pseudomonas* sp. MPC6 also contains the complete genetic machinery for the transport and mineralization of ethylene glycol, an important product of the enzymatic and thermal breakdown of Polyethylene terephthalate (PET) (Kenny et al., 2012; Yoshida et al., 2016).

With regards to the catabolism of aromatic compounds, the Antarctic MPC6 strain is able to convert a myriad of toxic compounds. It can transform phenol to catechol by the action of various phenol hydroxylases (P1, P2, and P5). Subsequently, catechol can replenish both the *ortho*- and *meta*-cleavage pathways as both aromatic routes are encoded in the chromosome of this Antarctic microbe, where the first route yields acetyl-CoA and succinyl-CoA and the latter pyruvate and acetyl-CoA. We also found protein-coding genes for the degradation of benzene and toluene such as the toluene 4-monooxygenase and toluene 4-sulfonate monooxygenase system, in addition to efflux pumps (TtgF enzymes) for cellular detoxification. Styrene, a starting material for the industrial polymer production and a widespread contaminant agent (O’Leary et al., 2002), may also be degraded by MPC6 given that it displays a styrene monooxygenase enzyme (encoded by the *styA* gene) and the genes encoding for enzymes of the side-chain oxidation pathway toward phenyl-acetic acid (PAA). This cold-adapted bacterium has various nitroreductase enzymes, which share a high amino acid sequence identity (92%) with the enzymes reported in other *Pseudomonas* bacteria able to reduce TNT (2,4,6-trinitritoluene). Genes responsible for the conversion of pentachlorophenol (PCP) were also found within the genome of *Pseudomonas* sp. MPC6, where the PCP 4-monooxygenase enzyme (encoded by *pcpB*) carries out the first conversion of PCP to tetrachloro-*p*-hydroquinone, which follows rings fission toward maleylacetate to fuel the TCA cycle via the ketoadipate pathway. Haloalkane compounds are another group of chemicals for which the MPC6 strain also displays the protein-coding genes for their catabolism. We identified three genes (*dhmA1*, *dhmA2*, and *dhaA*) encoding for haloalkane dehalogenase enzymes in the genome of *Pseudomonas* MPC6, which catalyze the hydrolytic conversion of chloroalkene and nitroalkane to the corresponding alcohol and hydrogen halide (Janssen, 2004). The byphenyl gene cluster *bphABCD*, encoding for enzymes catalyzing the mineralization of byphenyl to benzoic acid and 2-hydroxypenta-2,4-dienoate, was also found in the genome of this versatile Antarctic microbe.

With respect to carbon storage and exopolymeric compounds in this psychrotolerant strain, we also inspected the alginate, rhamnolipids, and PHAs biosynthetic pathways. As with other *Pseudomonas* strains, MPC6 has the capacity to produce alginate from different biosynthetic pathways such as glucose, glycerol, and fructose (Figure 3). The genetic conformation is very similar to the one displayed by *P. aeruginosa* PAO1 (Supplementary Figure S1). Interestingly, the genome of *Pseudomonas* sp. MPC6 contains five copies of the *algJ* gene encoding for an alginate *o*-acetyltransferase, an important enzyme that together with AlgF and AlgI form a membrane complex for the correct polymerization and transport of the exopolysaccharide along with the addition of acetyl groups to the chemical structure of alginate. Another class of metabolites that can be synthesized from one of the precursors of the alginate pathway are the biosurfactant glycolypids known as rhamnolipids, where the *algC* gene (encoding for a phosphoglucomutase) initiates the conversion of glucose 6-phosphate to dTDP-L-rhamnose (Chong and Li, 2017). This catalytic process is carried out by the enzymes RmlA, B, C, D, and all of them are encoded in *Pseudomonas* sp. MPC6 genome. The final enzymatic steps for rhamnolipid synthesis are the dimerization of 3-hydroacyl-ACP (acyl carrier protein) to 3(3-hydroxyalkanolyoxy)alkanoate (HAA) and the condensation of dTDP-L-rhamnose and HAA, mediated by RhlA and RhlB, respectively. The MPC6 strain possesses the RhlA enzyme but does not contain open reading frames encoding for the RhlB enzyme (rhamnosyltransferase I), thus prohibiting the last reaction for rhamnolipid synthesis. Besides alginate, another EPS related genes were found in the chromosome of MPC6, where *pga*A and B encode a Poly-beta-1,6-*N*-acetyl-D-glucosamine export protein and Poly-beta-1,6-*N*-acetyl-D-glucosamine *N*-deacetylase, respectively, both required for biofilm formation (Wang et al., 2004).

Regarding the production of polymeric substances, in a recent study (Pacheco et al., 2019) we showed that *Pseudomonas* sp. MPC6 synthesizes a unique copolymer of short- and medium-chain length PHAs on glycerol. Surprisingly, the genome of MPC6 strain encodes five PHA synthases (PhaC) and can generate precursors from the condensation of acetyl-CoA to acetoacetyl-CoA which is further reduced to (R)-3-hydrobutyryl-CoA by the PhaB enzyme-the main pathway for the synthesis of Poly(3-hydroxybutyrate) (PHB) (Madison and Huisman, 1999). PHA synthesis can also proceed from the synthesis *de novo* fatty acid route, where the (R)-3-hydroxyacyl-ACP moieties are converted to (R)-3-hydroxyacyl-CoA by PhaG (3-hydroxyacyl-ACP transferase), creating a link between the synthesis of fatty acids and PHAs (Rehm et al., 2001). Both pathways have been described as working simultaneously in bacterial strains engineered for PHA synthesis (Tappel et al., 2012). These results attest to the high capacity of *Pseudomonas* sp. MPC6 to synthesize and accumulate exopolysacchrides and inclusion bodies as a reservoir of carbon and energy, respectively, under fluctuating nutrient conditions and cold environments.

### Growth Validation on Various Carbon Sources and Temperature Response of *Pseudomonas* sp. MPC6 on Glycerol

**FIGURE 4:**
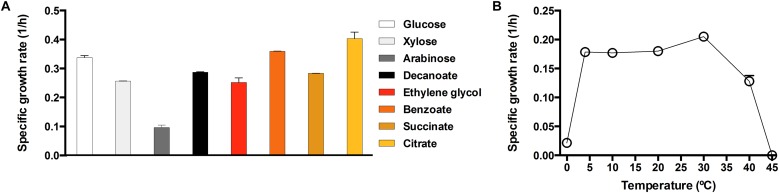
Specific growth rates of the strain *Pseudomonas* sp. MPC6. Specific growth rate (1/h) of the psychrotolerant bacterium in presence of various carbon sources **(A)**, or at different temperatures on glycerol **(B)**. Each experiment was conducted at least in triplicates. Error bars represent the standard deviation.

In order to fully validate the catabolic capabilities of the Antarctic MPC6 strain on the use of various C sources, we grew this bacterium on minimal medium in shaking flask experiments recording the change of Ln(OD_600_) over time to obtain specific growth rates (μ_max_) of the used carbon substrates (Figure 4A). The highest μ_max_ was obtained for citrate and benzoate (Figure 4A), the preferred carbon sources for *Pseudomonas* strains (Rojo, 2010). In addition, it was able to grow in the C5 carbon sugars xylose and arabinose, showing a μ_max_ of 0.26 and 0.10 (1/h), respectively. Another important finding is that *Pseudomonas* sp. MPC6 could efficiently propagate on ethylene glycol as the only C source, a feature which to date, has only been reported for *Pseudomonas putida* JM37 (Mückschel et al., 2012), where the Antarctic strain showed a growth rate of 0.25 (1/h) (Figure 4A).

We next evaluated the growth rate of MPC6 across a wide range of temperature (from 0 to 45°C) on glycerol. It is worth mentioning that for the measurement at 0°C, the glycerol concentration was increased from 4 to 30 (g/L) to avoid the water reaching its freezing point in the bioreactor. As depicted in Figure 4B, *Pseudomonas* sp. MPC6 grows at an optimum temperature of 30°C (0.21 1/h) from glycerol, whereas at 45°C the Antarctic microbe was no longer able to propagate. The growth rate showed a high reduction (∼10 times) when cells were cultured at 0°C as compared to the cells growing at optimal temperature (Figure 4B). Several studies have reported that psychrophilic microbes possessing enhanced ratios of lysine/arginine (K/R) or reduced arginine or proline contents are better at surviving extreme cold-temperatures (Methé et al., 2005; De Maayer et al., 2014). Inspection of the amino acid content from the genome of MPC6 reveals that there is no variation in the K/R ratio as compared to that of *P. putida* KT2440 (mesophile) but shows an elevated proline content (Supplementary Table S3).

### Virulence and Antibiotic Resistance Profile

**FIGURE 5:**
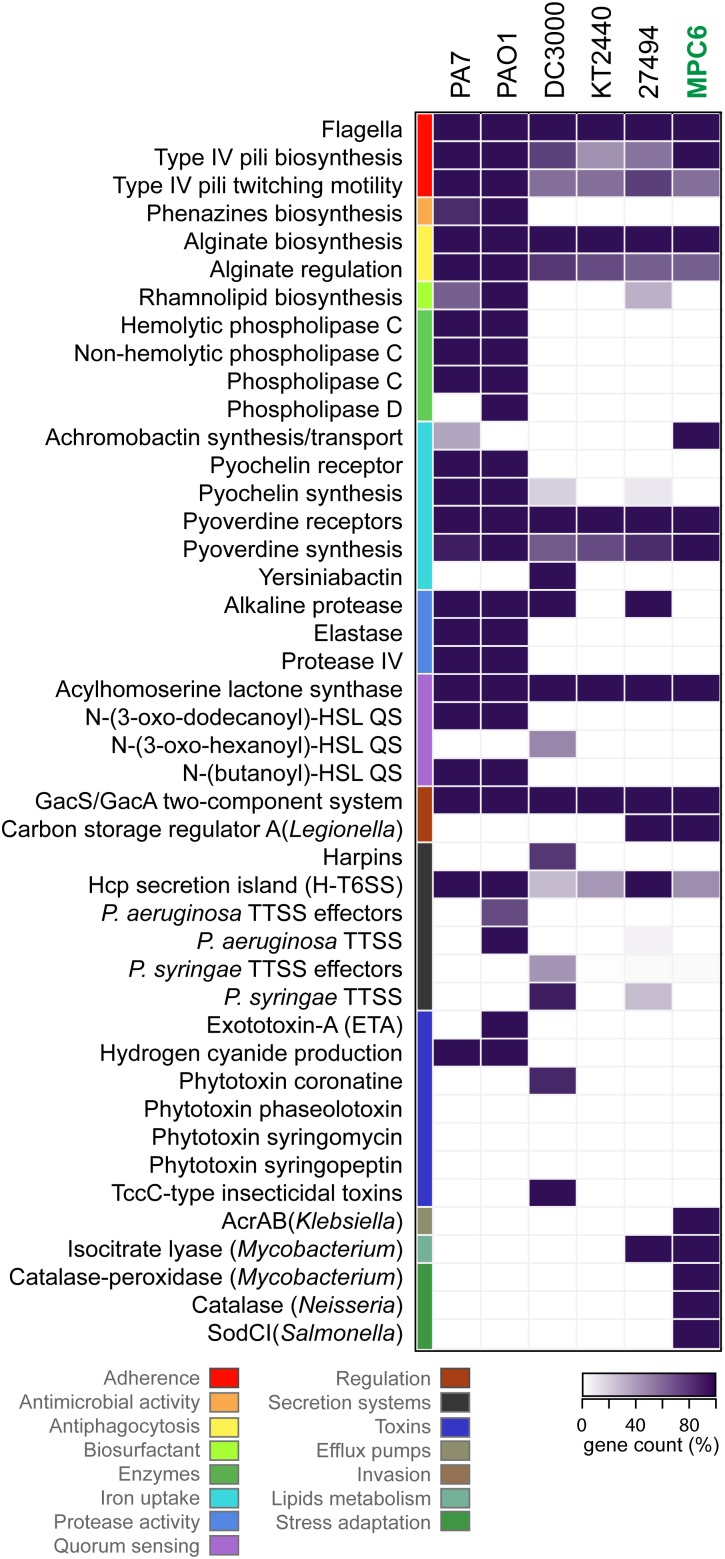
Genome virulence profile of *Pseudomonas* sp. MPC6 and other pathogenic and non-pathogenic *Pseudomonas* strains. Putative virulence-associated genes encoded in the genome of each strain were identified, classified and counted using the resources available at VFDB and PATRIC databases. Virulence factors were grouped into functional categories that are depicted with different colors. The gene count for each category in each strain was plotted as a heatmap, were the darkest tone (100%) corresponds to the highest number of genes found in each category among all the strains.

In order to explore the safety and pathogenic potential (Casadevall, 2017) of *Pseudomonas* sp. MPC6, we analyzed its genome using the gene classification tools available at the VFDB and PATRIC virulence factors databases. We identified, classified and counted putative virulence-related genes, and compared them with those of other *Pseudomonas* strains previously described as non-pathogenic environmental isolates (*P. putida* KT2440, *P. antarctica* PAMC 27494), isolates virulent to mammals (*P. aeruginosa* PAO1, *P. aeruginosa* PA7), or virulent to plants (*P. syringae* DC3000) (Figure 5). The gene count for different categories of virulence factors showed marked differences among virulent strains and environmental isolates, where PA7 and PAO1 strains showed higher counts in most of the categories. Key virulence factors for infection of animals present in these strains are absent in *Pseudomonas* sp. MPC6 (as well as in *P. putida* KT2440, and *P. antartica*), including the *P. aeruginosa* type 3 secretion system and its effectors, several phospholipases, the elastase and protease IV enzymes, genes for production of phenazines, the exotoxin-A, two quorum sensing systems, and genes for the synthesis and uptake of the pyochelin siderophore. We also found that the MPC6 strain lacked important factors required for virulence over plants and insects that are present in *P. syringae* DC3000, including genes for producing the phytotoxin coronatine, the TccC-type insecticidal toxins, the *P. syringae* type 3 secretion system and its effectors, genes for producing the yersiniabactin siderophore, and for the production of harpins amyloid fibers involved in plant tissue colonization and biofilm formation. For comprehensive reviews of *P. aeruginosa* and *P. syringae* virulence factors please refer to Jimenez et al. (2012), Gellatly and Hancock (2013), and (Xin et al., 2018), respectively. On the other hand, the MPC6 genome encodes functions related to virulence that are absent or less represented in other strains, including the synthesis and transport of the siderophore achromobactin, an efflux pump from the AcrAB family, and enzymes required for stress adaptation and ROS detoxification such as a catalase, a catalase-peroxidase, and a superoxide dismutase. Although those genes were classified as virulence factors in the databases used, they can be classified in a more general way as factors for resilience to harsh environmental conditions either inside or outside a host organism.

**FIGURE 6:**
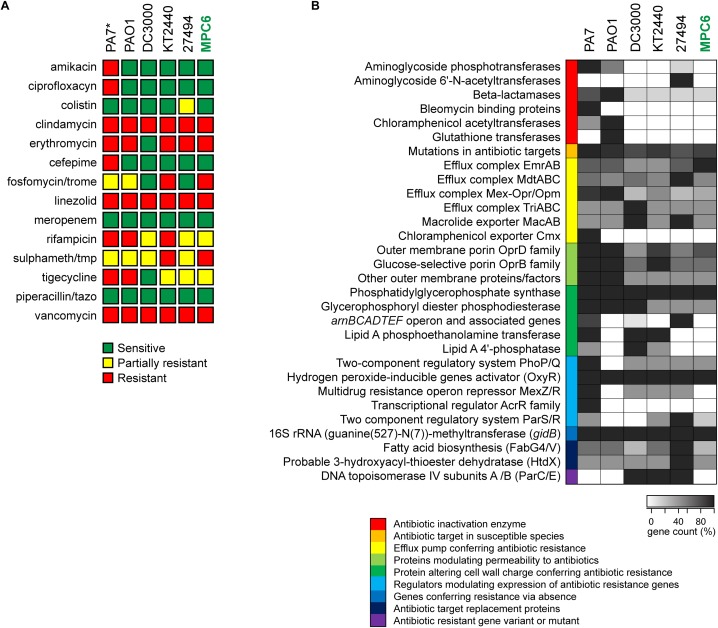
Antimicrobial resistance properties of *Pseudomonas* sp. MPC6 compared to other strains from this genus. **(A)** Susceptibility to different classes of antibiotics determined by disk diffusion assays. ^∗^The profile shown for *P. aeruginosa* PA7 was not determined experimentally in this work and was built based on previous studies. **(B)** Antimicrobial resistance profile determined based on genomic analyses. Putative factors associated to drug resistance were grouped into different categories that are depicted with different colors. The gene count for each category in each strain was plotted as a heatmap, were the darkest tone (100%) corresponds to the highest number of genes found in each category among all strains.

Additionally, we explored the MPC6 antimicrobial resistance profile both experimentally and *in silico* using the information from the genome (Figure 6). Disk diffusion assays to test the sensitivity to fourteen antibiotics from different families were performed for MPC6 as well as for PAO1, DC3000, KT2440, and *P. antarctica* (Figure 6A). Additionally, since we had no access to the PA7 strain, a bacterium well characterized as multiresistant, its sensitivity to each class of antibiotic was assigned based on assays reported in the literature (Köhler et al., 1996; Roy et al., 2010; Morita et al., 2012; Sader et al., 2018). As expected, PA7 was resistant to the highest number of antibiotics, including amikacin, ciprofloxacin and cefepime, which were effective over all the *Pseudomonas* strains tested. Conversely, *Pseudomonas* MPC6 was sensitive to antibiotics that were ineffective for most of the other strains, showing a similar resistant profile than the environmental isolates KT2440 and *P. antartica*. Of note, *P. syringae* DC3000 was shown to be sensitive to most of the antimicrobials.

Computational analyses using genomic data along with the databases CARD and PATRIC, allowed us to determine the resistome of MPC6 and the rest of the *Pseudomonas* strains, classifying and counting genes related to different molecular mechanisms leading to antimicrobial resistance (Figure 6B). In agreement with what was observed experimentally, MPC6 showed a similar or reduced load of drug resistance genes than the other environmental isolates, lacking several antibiotic inactivation enzymes present in PA7 or PAO1, including aminoglycoside phosphotransferases, chloramphenicol acetyltransferases, bleomycin binding proteins, and beta-lactamases. Moreover, the MPC6 strain lacked several genes related to alterations in the cell wall charge reported to confer resistance to antibiotics. On the other hand, the MPC6 genome seems to be well equipped with several efflux complexes and exporters, which could help it to cope with the toxic compounds present in its natural environment. Taken together, all this evidence indicates that *Pseudomonas* MPC6 has a low pathogenic potential and an antimicrobial resistance profile comparable to other non-virulent *Pseudomonas* environmental isolates. This supports the safety of *Pseudomonas* MPC6 for its usage as a valuable biotechnological tool.

### Resistance to Heavy Metals and Oxidative Stress

**FIGURE 7:**
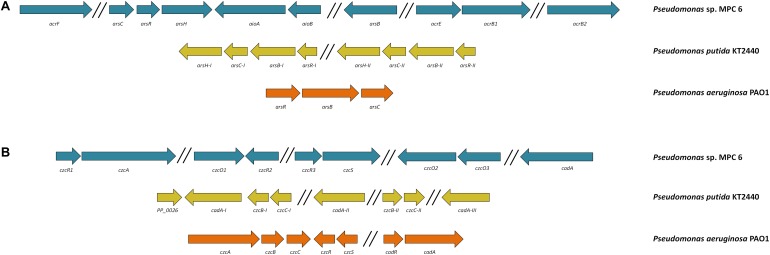
Genetic organization of **(A)** arsenic and **(B)** cadmium resistance genes of the Antarctic *Pseudomonas* sp. MPC6, KT2440, and PAO1.

Genome examination of strain MPC6 also revealed the presence of several genes reported to oxidize/reduce and to diminish the toxicity of arsenic and cadmium compounds. Their genetic conformation is displayed in Figure 7. This Antarctic bacterium can oxidize the most toxic metalloid arsenite As(III) to arsenate As(V) by the action of AsIII oxidases (*aioBA* encoding genes) (Figure 7A). It also holds the arsenate reductase genes *arsC* and *arsH* as well as the *arsAB* arsenite efflux complex, which allows to reduce arsenate to arsenite and pump it out of the cell, respectively. The multidrug efflux pump complex AcrAB was also found in MPC6, which would play an important role in bacterial metal detoxification (Yu et al., 2003). Concerning cadmium resistance genes, the internal membrane CzcA efflux cation-antiporter system was identified along with the CadA enzyme, where the latter is similar to the Cd-transporter ATPase system widely found in Gram-positive bacteria (Chellaiah, 2018). Remarkably, three activator genes (*czcR*) are together with different cadmium-resistance genes distributed in various regions of the genome (Figure 7B). Moreover, together with the sensor *czcS* gene, the *czcR* gene form a two component regulatory system (Perron et al., 2004). We also found three genes (*czcO*1, 2, 3) encoding for cation diffusion facilitator enzymes, which have been implicated in the export of metals in bacteria (Wei et al., 2006).

Given the high amount and diversity of genes related to metal resistance discovered in the Antarctic strain MPC6, we next evaluated the MIC for Cd, As, Cu, Hg, and further compared the obtained values with those from bacterial strains reported as highly resistant to metals (Table 1). To the best of our knowledge, *Pseudomonas* sp. MPC6 shows the highest MIC so far reported against AsV (350 mM) among *Pseudomonas* strains. It can also grow in the presence of high concentration of copper and AsIII, showing elevated resistance to cadmium (10 mM) as compared to other environmental *Pseudomonas*. Conversely, *Pseudomonas* sp. MPC6 showed to be highly sensitive to mercury (Table 1). It is well reported that psychrotolerant microbes have developed several molecular mechanisms to cope with cold environments and agents such as heavy metals, that exacerbate oxidative stress within the cell (Chattopadhyay et al., 2011). In this regard, the *Pseudomonas* sp. MPC6 genome encodes homologs to several known proteins related to oxidative stress alleviation including three catalases, three superoxide dismutase (Mn/Fe, Cu/Zn, Fe), two copies of glyoxalase ElbB, four glutathione *S*-transferases, three thioredoxins, pioredoxins, and more than twenty glutathione-related proteins (Table 2). In addition, various copies of genes encoding for cold and heat shock proteins were also detected, which would correspond to proteins that bind to RNA and DNA, facilitating the correct transcription and translation processes under cold conditions (Keto-Timonen et al., 2016).

**Table 1:** Minimum inhibitory concentration of metals in *Pseudomonas* sp. MPC6 and highly metal resistant *Pseudomonas* strains.

Metal	MIC (mM)	Strain	References
As(III)	10	*Pseudomonas* sp. MPC6	This study
As(V)	350	*Pseudomonas* sp. MPC6	This study
Mercury	0.015	*Pseudomonas* sp. MPC6	This study
Cadmium	10	*Pseudomonas* sp. MPC6	This study
Copper	6	*Pseudomonas* sp. MPC6	This study
As(III)	50	*Pseudomonas* sp. RJB-2	Banerjee et al., 2011
As(V)	250	*P. putida* KT2440	Fernández et al., 2014
Mercury	9.5	*P. fluorescens* 3	Roy and Nair, 2007
Copper	16	*P. aeruginosa* PAO1	Teitzel et al., 2006
Cadmium	7.6	*Pseudomonas* sp. Pse3	Plaza et al., 2016


**Table 2:** Genes involved in cold adaptation and oxidative stress found in *Pseudomonas* sp. MPC6.

Category and gene	Locus tag	Function/Description
*Cold shock proteins*		
*cspA_1, cspA_2, capB_1, capB_2, cspD*	MPC6_1161, MPC6_4988, MPC6_1237,	Cold shock proteins
	MPC6_2014, MPC6_4244	
*Heat shock proteins*		
*hslR, ibpA*	MPC6_360, MPC_1942	Heat shock proteins
*Oxidative stress*		
*cat, katE, katG*	MPC6_5660, MPC6_198, MPC6_2528	Catalase
*srpA, tpx*	MPC6_1816, MPC6_3164	Peroxidase
*ahpF, ahpC_1, ahpC_2*	MPC6_3361, MPC6_5109, MPC6_3360	Alkyl hydroperoxide reductase
*sodB_1, sodB_2, sodC*	MPC6_5073, MPC6_5372, MPC6_2518	Superoxide dismutase [Cu-Zn]/[Fe]/[Mn/Fe]
*trxA, trxC, btuE*	MPC6_6144, MPC6_530, MPC6_5341	Thioredoxin
*ggt_1, ggt_2, gloB_1, gloB_2, gloB_3, gloB_4, gloC*	MPC6_936, MPC6_4218, MPC6_3235	Glutathione hydrolase
	*MPC6_3293, MPC6_3654, MPC6_4725, MPC6_988*	
*frmA_1, frmA_2, frmA_3*	MPC6_1199, MPC6_3480, MPC6_4183	Glutathione dehydrogenase
*yfcF, gstB_1, gstB_2, MPC6_06172*	MPC6_5240, MPC6_1445, MPC6_4938, MPC6_6172	Glutathione *S*-transferase
*gsiC_1, gsiC_2, gsiC_3, gsiD_1, gsiD_2, gsiD_3*	MPC6_2267, MPC6_4121, MPC6_5806,	Glutathione permease
	*MPC6_2268, MPC6_4120, MPC6_5807*	
*gsiA_1, gsiA_2*	MPC6_2269, MPC6_5808	Glutathione import ATP-binding protein
*gshAB*	MPC6_1518, MPC6_6004	Glutathione synthetase
*garB*	MPC6_3364	Glutathione reductase


### PHA Production Profile on Various Carbon Substrates

**FIGURE 8:**
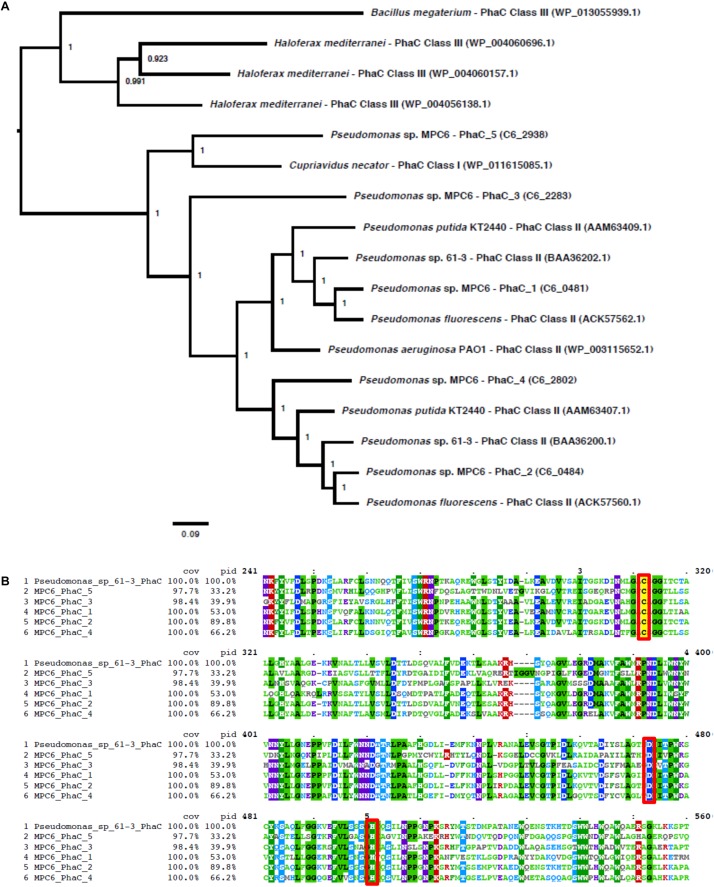
PhaC synthases comparison of various PHA-producing microbes. **(A)** Phylogenetic three of different PhaC synthases. **(B)** Amino acid sequence alignment of various PhaC synthases.

We have recently produced a novel *scl-*co-*mcl*-PHA from glycerol in *Pseudomonas* sp. MPC6 (Pacheco et al., 2019). In this context and considering the large genetic repertory coding for PHA-related enzymes displayed by MPC6, we performed batch cultures in flasks to quantify and characterize via GC/MS the biosynthesized PHAs from different carbon sources. Using glucose as C source, the MPC6 strain produced high levels of *scl*-co-*mcl*-PHA (∼1.7 g/L), accumulating more than 30% of the CDM as PHA. Surprisingly, xylose- and arabinose-grown MPC6 cells synthesized only *mcl*-PHA consisting of monomers of 3-hydroxyhexanoate (C6), 3-hydroxyoctanoate (C8), 3-hydroxydecanoate (C10), and 3-hydroxydodecanoate (C12) (Table 3). The same monomer composition was determined for PHAs synthesized from ethylene glycol, where the major monomer was C10, making the first time that this biopolymer is produced by a wild-type *Pseudomonas* strain using ethylene glycol as the solely C substrate. A little portion of the monomer 3-hydroxybutyrate (C4) was identified in the biosynthesized *scl*-co-*mcl*-PHAs, where only 2.1 and 3.2% of the polymeric chain was identified as C4 when succinate and citrate were employed as substrates, respectively (Table 3). As the PHA synthase defines, to a great extent, the final monomer composition of the produced PHA, we compared the five PHA synthases of *Pseudomonas* sp. MPC6 against the polymerase enzymes (PhaC) of well-studied PHA producing microbes like *Bacillus megaterium*, *Haloferax mediterranei*, *Cupriavidus necator*, *P. putida* KT2440, and *Pseudomonas* sp. 61-3, the latter bacterium being the only *Pseudomonas* strain described to be capable of naturally producing scl-*co*-mcl-PHAs. Based on the amino acid sequence identity of the studied PHA synthases, phylogenetic analysis revealed that out of the five PHA synthases, one of them (PhaC5) cluster with the Class I synthases of *C. necator*, whereas PhaC1, PhaC2, and PhaC4 clustered closer to Class II synthases of *P. putida* KT2440 and *Pseudomonas* sp. 61-3 (Figure 8A). The PhaC3 of *Pseudomonas* sp. MPC6 was more distantly related from the other Class II synthases, positioning itself in a separated branch of the tree. The multiple amino acid sequence alignment corroborates the fact that PhaC3 differs from the PhaC polymerase of *C. necator*, *P. putida* KT2440 and *Pseudomonas* sp. 61-3, showing 34.4, 40.4, and 39.9% amino acids sequence similarity, respectively (Figure 8B). Further molecular studies will need to be conducted in order to fully elucidate the role of each PHA synthase of the psychrotolerant MPC6 bacterium while producing PHAs on various carbon substrates.

**Table 3:** Monomer composition, biomass, and PHA titers displayed by *Pseudomonas* sp. MPC6 in shaking flask experiments after 96 h cultivation.

C source (g/L)	CDM (g/L)^a^	PHA (%)^b^	PHA (g/L)^a^	Monomer composition (%)^c^					
				
				C4	C6	C8	C10	C12	C12:1
Glucose (30)	5.45 ± 0.2	31.5	1.72 ± 0.1	81.6	0.1	6.0	12.3	N.D	N.D
Xylose (30)	4.62 ± 0.1	3.8	0.15 ± 0.0	N.D	11	16.6	66.1	6.3	N.D
Arabinose (30)	1.42 ± 0.1	8.9	0.13 ± 0.1	N.D	6.1	23.9	45.9	24.2	N.D
Ethylene glycol (30)	1.37 ± 0.0	15.5	0.21 ± 0.0	N.D	19.5	40.3	40.2	N.D	N.D
Decanoate (4)	2.61 ± 0.0	36.8	0.65 ± 0.1	1.3	3.3	55.3	40.1	N.D	N.D
Succinate (30)	1.74 ± 0.0	22.4	0.39 ± 0.1	2.1	2.5	32.8	54.6	6.2	1.7
Citrate (30)	1.75 ± 0.1	18.9	0.33 ± 0.0	3.2	2.6	21.4	62.8	5.4	4.6


*^*a*^Values represent means and (±) SD from at least three independent experiments. ^*b*^PHA content relative to cell dry mass (CDM). ^*c*^C4: 3-hydroxybutyrate, C6: 3-hydroxyhexanoate, C8: 3-hydroxyoctanoate, C10: 3-hydroxydecanoate, C12: 3-hydroxydodecanoate, C12:1: 3-hydroxy-5-cis-dodecanoate. N.D, not detected.*

## Discussion

Today, the genomes of more than 180 psychrophiles have been sequenced providing a vast genetic inventory of psychrophilic and psychrotolerant microorganisms (Bowman, 2017), yet much remains to be learned about the role of a significant number of genes with no predicted function and how they contribute to the successful lifestyle of these microbes at low temperatures. Connecting genes with functions and the resulting phenotypes of psychrotolerant bacteria remains one of the biggest challenges in biology (Walhout et al., 2011). Cold-adapted bacteria are well characterized for their ability to optimally propagate at temperatures above 25°C and displaying cell division at 4°C, but with a highly reduced growth rate (10-fold) (Ayub et al., 2004; Fondi et al., 2015). At low temperatures, DNA replication, protein synthesis, and enzyme catalytic efficiency are reduced (D’Amico et al., 2006; Feller, 2013), the latter due to thermodynamic constraints. This negatively impacts substrate uptake rates and metabolic fluxes, ultimately resulting in resource reallocation within the cell (Knoblauch et al., 1999; Czajka et al., 2018). In-depth analysis of the genome of the *Pseudomonas* sp. MPC6 strain, which is able to grow at nearly the same rate and produce the same amount of biomass under a wide range of temperatures (Pacheco et al., 2019), could shed lights on the genetic components that are necessary for endowing bacteria with the means to thrive rapidly when exposed to low temperatures. First, it is clear that microorganisms that encounter cyclic fluctuation of temperature, such as those inhabiting the soil of North Antarctic tundra, display phenotypes features that differ from those observed in microbes permanently exposed to temperatures below 4°C -as in sea water and permafrost environments (De Maayer et al., 2014).

As most of the Antarctic territory has not been explored with the aim of isolating bacterial strains, novel species from all genera of bacteria must inhabit this pristine environment. In this respect, phylogenetic analysis using whole genome data strongly suggests that *Pseudomonas* sp. MPC6 should be classified as a new species inside this genus (Figure 2). Here, we propose the name *Pseudomonas frigusceleri* MPC6, due to its outstanding ability to proliferate and produce valuable compounds at temperatures near to 0°C. Furthermore, this isolate clustered inside the *Pseudomonas fluorescens* phylogroup, which encompasses most of the *Pseudomonas* environmental strains described so far, isolated from very diverse habitats. However, several of the MPC6 closest relatives belonged to a variety of species that also appeared intermingled across other branches inside this phylogroup. This points to the extreme diversity of bacteria classified as *Pseudomonas*, one of the most extensive bacterial genera described so far comprising more than 200 species (Gomila et al., 2015; Peix et al., 2018). In addition, this indicates that the classification at the species level inside this genus seems to contain conflicts for several strains and needs further revision. Approaches taking advantage of genomic data such as the one presented here allow to conduct this task with higher resolution than those based only on the analysis of the 16S rDNA or the classical typing scheme using six *loci*.

As reported for several bacterial strains of the genus, *Pseudomonas* sp. MPC6 is metabolically versatile since it can metabolize a myriad of carbon compounds and use them as the unique carbon and energy source (Figure 3, 4). However, *Pseudomonas* strains isolated so far from Antarctic territory have not shown these metabolic capabilities, and even though they can metabolize various C6 carbon sugars, none of them are able to catabolize xylose or arabinose (Lustman et al., 2015; Lee et al., 2017). Most likely, the MPC6 strain has evolved the genetic repertory to metabolize these sugars (C6 and C5), glycerol, organic acids, and aromatic compounds in part due to the fact that Deception Island, where this bacterium was isolated, has one of the largest reservoir of the Antarctic flowering plants *Deschampsia Antarctica* and *Colobanthus quitensis* (Komárková et al., 1985); self-fertilizing species that grow as cushions and mats, respectively (Cho et al., 2018) and might provide these carbon substrates. By analyzing the particular environmental and geographical conditions present at the Deception Island, it is possible to decipher and give a concise explanation of the observed genetic traits of strain MPC6. As a result of the intense volcanic activity -the last back in 1970 (Higham et al., 1984)- the levels of heavy metals such as Cd, As, and Pb at Deception Island have been recorded among the highest within the Antarctic territory (Mão de Ferro et al., 2013). To cope with the elevated concentration of metals, the MPC6 bacterium must indeed oxidize/reduce both states of arsenic compounds to alleviate toxicity accordingly. Few *Pseudomonas* bacteria have been reported to harbor genes to oxidize As(III) to As(V) (Ghosh et al., 2015), where both models strains *P. putida* KT2440 and *P. aeruginosa* PAO1 are only able to reduce or expel arsenate(V) from the cell (Fernández et al., 2016; Fekih et al., 2018), the most abundant arsenic form in nature. It has been reported that As-resistant bacteria are dependent on their capacity to carry out oxidation or reduction for their survival to arsenic compounds (Ghosh et al., 2015), thus strain MPC6 has a more efficient mechanism toward As(V) reduction given its outstanding capability to withstand arsenate (Table 1). The high copy number of genes found in MPC6 implicated in fighting oxidative stress (catalases, superoxide dismutase, oxidoreductases) (Table 2), might also contribute to its survival. Moreover, *Pseudomonas* sp. MPC6 can also be described as a multi-metal-resistant strain since it is capable of growing in the presence of high levels of cadmium (10 mM). Microbes exposed to concentrations of 3 mM of Cd^2+^ are already classified as resistant strains (Higham et al., 1984). Bacteria of the *Pseudomonas* genus normally display a mechanism where cadmium is first accumulated within the cell (Lee et al., 2001), and its toxicity is further reduced by the action of polyphosphates and cysteine-rich proteins (Higham et al., 1984; Wang and Crowley, 2005). Beside these genetic mechanisms, the MPC6 bacterium possess several *czc* genes responsible for exporting cadmium to the extracellular environment (Hu and Zhao, 2007) along with more than 20 glutathione-related proteins, which are also involved in metal-binding resistance of cellular thiols (Díaz-Cruz et al., 1997; Helbig et al., 2008). Another important finding concerns the high number of genes belonging to the catabolism of toxic chloro- and nitro-aromatic, aromatic, and alkanes compounds, some of which have been described as originating from anthropogenic activities (Kunze et al., 2009; Ju and Parales, 2010). Accordingly, Deception Island is one of the most visited zones of the Antarctic South Shetland archipelago (more than 20,000 people per year) and served as a trading point for more than two decades due to a whaling factory and tourism (Killingbeck, 1977; Valero-Navarro et al., 2007). In addition, there are several military bases where the use of hydrocarbons is constantly needed (Valero-Navarro et al., 2007). As the handling of fuels and hydrocarbons in Deception was not regulated until 2005, several spills may have occurred in the past. Likely, the human footprint along with hydrocarbons and heavy metals emanating from volcanic eruptions at Deception have exerted a selective pressure on the MPC6 microbe, driving its evolution toward multiple pathways for degradation of aromatic compounds and resilience to harsh environmental conditions.

One of the most remarkable characteristics of *Pseudomonas* sp. MPC6 strain corresponds to its ability to grow close to μ_max_ (0.21 1/h) for a wide range of temperatures (Figure 4B). At temperatures below 10°C, growth of psychrotolerant microbes is usually highly arrested (Ayub et al., 2004; Fondi et al., 2015). There are several geothermal sites across the Deception island with temperatures reaching as much as 98°C (Muñoz et al., 2011), creating microenvironments with a gradient of temperature in the surroundings area that sustain different forms of life (Bendia et al., 2018). Taking this into account, the MPC6 strain has evolved to colonize those niches with fluctuating temperatures, but what genetic means makes this versatile bacterium so different from other cold-adapted *Pseudomonas* bacteria? It has been proposed that high copy number of tRNAs contribute to the survival of psychrophiles in response to low temperatures (Methé et al., 2005). We excluded this possibility in MPC6 since the numbers of tRNAs found in this Antarctic bacteria do not differ from those observed in mesophilic *Pseudomonas* strains (Nelson et al., 2002; Vizoso et al., 2015). Additionally, the unaltered ratio of lysine/arginine and enhanced proline content found in the genome of *Pseudomonas* sp. MPC6 in comparison to mesophilic *Pseudomonas* indicates that other genetic elements govern the adaptation of the MPC6 strain under low temperatures. On the other hand, we showed that *Pseudomonas* sp. MPC6 harbors three novel plasmids (Figure 1B), comprising in total more than 380 genes of diverse function. Of note, two of these plasmids seem to be conjugative, suggesting that horizontal gene transfer would be an important contributor to genetic variation and diversity, even in this kind of extreme environments. Among the plasmid-encoded functions, we identified genes putatively involved in the metabolism, uptake and efflux of a plethora of compounds, including a remarkable number of transporters, oxidoreductases, and transcriptional regulators, likely contributing to the stress resilience and metabolic versatility of this extremophile. In view of the experimental and computational evidence provided here, we propose that the diverse genetic repertoire participating on a variety of cellular processes enables strain MPC6 to replicate at a high rate at cold-temperatures. This property would involve the synthesis of EPS, the expression of a high number of genes related to the response to cold shock and ROS, a high-level intracellular production of PHAs, and the activity of several transport systems for L-carnitine, trehalose, and betain, among other compounds (Figure 3). In order to rank each of these cold-responsive mechanisms according to their functional importance at various molecular levels, further functional analyses using transcriptomics, proteomics, and metabolomics are required. It is clear that the interrelationship of these molecular layers allows MPC6 strain to maintain metabolic functions under a wide range of temperatures.

Based on the results of this study, we conclude that the new species of pseudomonad, *Pseudomonas frigusceleri* MPC6 strain, is genetically well equipped for cold adaptation, shows a reduced pathogenic and antibiotic resistance potential, and exhibits attributes that hold promising biotechnological applications such as bioremediation at low temperature of arsenic, cadmium, alkanes, and chloro- and nitro-aromatics compounds, as well as the synthesis of biopolymers such as alginate and novel PHAs from renewable sources.

## Author Contributions

IP-C and AM conceived the study. MO-S, MM, and NP performed the genome analysis and growth performance of the MPC6 strain at different temperatures and on various C substrates, MIC assays, and PHA production experiments. AM, EC-N, CM, KM carried out the genome sequencing and assembly. JC and AM performed the core-genome MLSA phylogenetic analysis, the virulence factors analysis and the experimental and *in silico* antimicrobial sensitivity profiling. IP-C and AM wrote the manuscript. All authors contributed with valuable discussions and edition, approving the final version of manuscript.

## Conflict of Interest Statement

The authors declare that the research was conducted in the absence of any commercial or financial relationships that could be construed as a potential conflict of interest.

## Footnotes

1 www.modelseed.org
