# Supplementary material for: In-Depth Genomic and Phenotypic Characterization of the Antarctic Psychrotolerant Strain Pseudomonas sp. MPC6 Reveals Unique Metabolic Features, Plasticity, and Biotechnological Potential
Source: Front Microbiol. 2019 May 24;10:1154. doi: 10.3389/fmicb.2019.01154 (PMC6543543; doi:10.3389/fmicb.2019.01154)
Supplement: Supplementary file 1 [file Table_1.DOCX]

**Supplementary Table 1.** Main genomic features of *Pseudomonas* sp. MPC6.

| Replicon | Genbank accession | Size (bp) | GC content | Protein coding genes | Other encoded features |
| --- | --- | --- | --- | --- | --- |
| chromosome | CP034783 | 6,841,168 | 59.96% | 6330 CDS | 69 tRNA-coding genes, 22 rRNA-coding genes, no CRISPR repeats |
| pMPC6-328K | CP034782 | 328,214 | 55.40% | 326 CDS | Type 4 secretion system, metabolism and uptake of L-carnitine, phenoxybenzoate, succinilbenzoate, gamma-aminobutyraldehyde, glycine, manganese, and zinc, among others |
| pMPC6-47K | CP034781 | 47,564 | 54.72% | 51 CDS | Type 4 secretion system, Type 1 restriction-modification system, tartrate/oxaloacetate metabolism |
| pMPC6-4K | CP034780 | 4,268 | 53.87% | 4 CDS | Mostly hypothetical proteins of unknown function |
