# Supplementary material for: In-Depth Genomic and Phenotypic Characterization of the Antarctic Psychrotolerant Strain Pseudomonas sp. MPC6 Reveals Unique Metabolic Features, Plasticity, and Biotechnological Potential
Source: Front Microbiol. 2019 May 24;10:1154. doi: 10.3389/fmicb.2019.01154 (PMC6543543; doi:10.3389/fmicb.2019.01154)
Supplement: Supplementary file 3 [file Table_3.DOCX]

**Supplementary Table 3.** Amino acid content of *Pseudomonas* sp. MPC6, *P. putida* KT2440 and *P. antarctica*.

| Amino Acid | Composition per proteome | | |
| --- | --- | --- | --- |
|  | *Pseudomonas putida* KT2440 | *Pseudomonas antarctica* | MPC6 |
| A | 0,1117 | 0,1096 | 0,1077 |
| C | 0,0658 | 0,0604 | 0,0621 |
| D | 0,0299 | 0,0321 | 0,0310 |
| E | 0,0534 | 0,0534 | 0,0535 |
| F | 0,0102 | 0,0095 | 0,0103 |
| G | 0,0567 | 0,0544 | 0,0559 |
| H | 0,0469 | 0,0454 | 0,0442 |
| I | 0,0802 | 0,0791 | 0,0796 |
| **K** | **0,0235** | **0,0228** | **0,0230** |
| L | 0,0459 | 0,0486 | 0,0502 |
| M | 0,1174 | 0,1166 | 0,1152 |
| N | 0,0340 | 0,0370 | 0,0363 |
| P | 0,0233 | 0,0231 | 0,0246 |
| Q | 0,0357 | 0,0367 | 0,0376 |
| **R** | **0,0488** | **0,0483** | **0,0487** |
| S | 0,0564 | 0,0588 | 0,0587 |
| T | 0,0479 | 0,0521 | 0,0501 |
| V | 0,0145 | 0,0143 | 0,0144 |
| W | 0,0256 | 0,0255 | 0,0256 |
| Y | 0,0722 | 0,0723 | 0,0714 |

*Amino Acid Composition=(Number of amino acid type X)/(Total number of amino acid)
