# Supplementary material for: In-Depth Genomic and Phenotypic Characterization of the Antarctic Psychrotolerant Strain Pseudomonas sp. MPC6 Reveals Unique Metabolic Features, Plasticity, and Biotechnological Potential
Source: Front Microbiol. 2019 May 24;10:1154. doi: 10.3389/fmicb.2019.01154 (PMC6543543; doi:10.3389/fmicb.2019.01154)

Figure S1. Genetic organization of (A) alginate and (B) PHAs biosynthetic pathways of the Antarctic *Pseudomonas* sp. MPC6 and other mesophilic *Pseudomonas* strains.

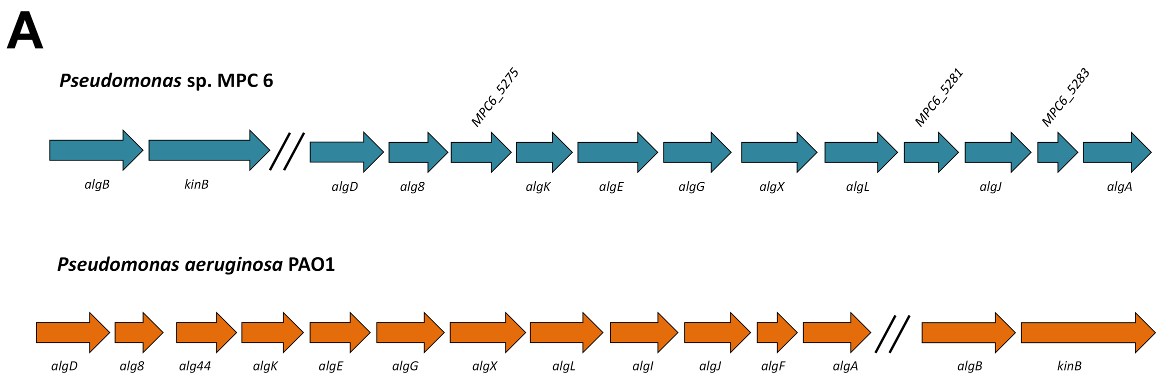

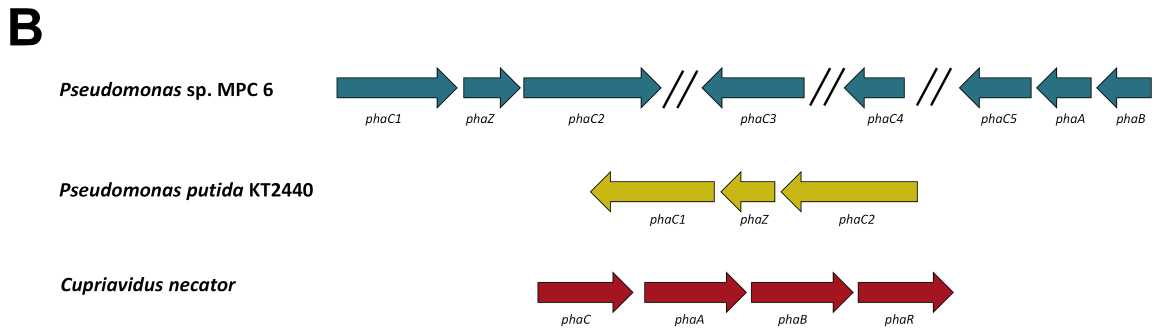

Supplement: Supplementary file 4 [file Data_Sheet_1.PDF]
